# Annotations

**Published:** 1895-03-02

**Authors:** 


### ANNOTATIONS.

### Infectious Diseases: The Fruits of Notification.

It is decidedly disquieting to be told by Mr. D. Biddle, who speaks with some authority on the subject, that, so far as he can judge, the "Notification Act has had no beneficial effect, except in regard to the diseases which are non-notifiable." That the Notification Act was well intended, and that it was based on theories the soundness of which no one now disputes, is generally admitted. Expectation was almost more than confident that its general adoption in every metropolitan district would lead to a gradual fall in zymotic diseases, especially, of course, of the notifiable class; and to a diminution in the death-rate from these diseases. Neither of these expectations has so far been justified. The number of cases in proportion to population has not lessened, nor has the removal of notified cases to the hospitals of the Asylums Board diminished the mortality. On the contrary, so far as those zymotic diseases which are notifiable are concerned, there has been an increase both in the number and in the fatality of the cases during the last quinquennium as compared with the quinquennium immediately preceding it. There may have been hidden forces at work of a temporary character to produce results so unwelcome and so unforeseen. In any case, no definite conclusions against notification are to be drawn from the comparison of only two quinquennia. The Act must be tried for an adequate period before any conclusions of an absolutely trustworthy character can be deduced from its working.

### John Hunter as a Biologist.

A certain melancholy interest attaches to the Hunterian oration, the last work undertaken by Mr. Hulke, read for him after he was taken ill, and published after he was dead; and all the more from the fact that this oration, written by a man of many parts, was itself devoted to showing how many-sided Hunter's work had been; that he was not merely a great anatomist, a great surgeon, or a great physiologist, but that he had extended his investigations widely through other branches of biology and that almost whatever he had seen he had made a subject of inquiry. Much as we have gained during this century in exactitude of knowledge, John Hunter was able by the mere use of his eyes, and by the deductions he drew from his observations, to foreshadow much that is now looked upon as the product of this century's investigations. The unity of the process of life whether in animal or in vegetable form; the essential difference between living and non-living matter; the different parts taken by vegetables and animals in the scheme of life; the fact that vegetables alone have the power of immediately converting "common" or inorganic matter into their own kind, and that, therefore, "a vegetable seems an intermediate link between common and animal matter," were all seen by him and were demonstrated as deductions from his own personal observations. Even the geological side of biology, and the significance of the traces still left of past forms of life, did not escape him; he studied and collected their remains, and, in a memoir presented to the Royal Society on a series of fossil bones, he investigated the circumstances of their fossilisation, compared their forms with those of extant animals, and from these and similar considerations, inferred a duration of our earth prolonged through "many thousand centuries." This, which is now looked upon as one of the ordinary axioms to be taught in elementary schools, was then regarded with so much misgiving, even by so learned a society, that the suggestion was conveyed to him that he should substitute years for centuries. He declined, however, to make the alteration, and the paper was withdrawn. Bees, dragon-flies, and beetles, everything which was endowed with life, was to Hunter a subject for inquiry and investigation, by observation so far as that would take him, and then further by experiment, if experiment were possible. A study of his life makes it stand out more clearly than ever that no man need complain of lack of opportunity or of subjects on which to exercise the mind; upon the smallest matters John Hunter turned the full illuminating power of his intellect, showing among other lessons that it is the mind and not the opportunity which makes the man.

### Influenza: Do Doctors Know Anything About It?

An evening contemporary assures its readers that notwithstanding the fact that we are now in the midst of the fifth successive annual epidemic of influenza, doctors know little or nothing about it. There is, perhaps, some justification for this in the circumstance that a good many immature practitioners, who desire to pose as scientists *in excelsis*, have assured the public on many occasions that science really cannot say what influenza is. But now let us ask ourselves with the downrightness of mere common sense what it is that our profession really does know, and know thoroughly, about influenza? In the first place, we know the disease when we see it; we know also the injurious physiological and pathological changes it produces in the nervous system, the lungs, the liver, and other organs of the body; we know how, by prompt early treatment, to reduce those changes to a minimum; and we know how to repair the damage done by those changes when the disease is brought to a termination. "But," it will be said, "if you claim to know all these things you claim to know everything about influenza?" No; we do not. We do not claim to know precisely what its cause is; nor do we profess to know entirely how to prevent it. But do we know what the cause of cancer is; or of typhoid fever; or of simple, or even of tubercular meningitis, and a hundred other things? Moreover, in the matter of prevention, can we prevent all other diseases of every kind except influenza? Can the lawyer, who thoroughly understands law, prevent crime? Can the theologian prevent sin? Can even the commercial man put an end to bankruptcy. Influenza has now been with us for five successive years. We can recognise it, we can treat it rationally and successfully; and to some extent we can prevent it. Perhaps when Providence has endowed us with omniscience and with almightiness as well, we may be able to entirely prevent the disease as well as to cure it. In the meantime, a little "silence" might be "golden" on the part of the all-knowing lay journalist.

